# Editorial: Resources for developing plasmid databases

**DOI:** 10.3389/fmicb.2025.1719919

**Published:** 2025-11-11

**Authors:** Haruo Suzuki, Masato Suzuki, Masaki Shintani

**Affiliations:** 1 Faculty of Environment and Information Studies, Keio University, Fujisawa, Kanagawa, Japan; 2 Antimicrobial Resistance Research Center, National Institute of Infectious Diseases, Japan Institute for Health Security, Tokyo, Japan; 3 Faculty of Engineering, Shizuoka University, Shizuoka, Japan; 4 Graduate School of Integrated Science and Technology, Shizuoka University, Shizuoka, Japan; 5 Research Institute of Green Science and Technology, Shizuoka University, Shizuoka, Japan; 6 Japan Collection of Microorganisms, RIKEN BioResource Research Center, Ibaraki, Japan

**Keywords:** plasmid, database, host, replication, antimicrobial resistance

Plasmids are extrachromosomal DNA elements that play pivotal roles in microbial metabolism, virulence, and antimicrobial resistance (AMR). By facilitating horizontal gene transfer, they drive rapid bacterial evolution and gene flow across diverse environments. Since the 1970s, plasmid studies have shaped our understanding of microbial genetics, with early pioneering work inspiring global efforts. Despite the vast accumulation of plasmid sequence data in international repositories, such as IMG/PR (Camargo et al., 2024) and PLSDB (Galata et al., 2018; Schmartz et al., 2022; Molano et al., 2024), existing databases suffer from misannotations, incompleteness, and data bias. Addressing these challenges requires a combination of expert knowledge, experimental validation, and innovative computational tools.

This Research Topic brings together contributions that span clinical case studies of AMR, evolutionary insights from environmental microbiology, and the development of bioinformatics methods and broad-host delivery vectors. Collectively, these works highlight both the urgency and opportunity to enhance the accuracy and utility of plasmid resources.

In the clinical domain, several studies underscore the continuing threat of plasmids carrying AMR genes, such as carbapenemase genes. A study on *Klebsiella pneumoniae* revealed IncX3 plasmids with *bla*_NDM − 1_ and a rare integron. In 1765, showing complex genetic architectures that complicate treatment options (Ma et al.). Complementary work on *K. pneumoniae* isolates from Pakistan revealed multiple plasmids encoding 34 resistance genes across six antimicrobial classes, suggesting intense plasmid exchange within a single hospital environment (Lascols et al.). Adding to this, the first report of *bla*_NDM − 1_-carrying *Enterobacter chengduensis* in China identified IncC plasmids and potential intrahospital clonal transmission (Fu et al.). Together, these case studies demonstrate how rapidly plasmids can disseminate AMR genes in diverse clinical settings, underscoring the urgent need for accurate plasmid characterization and surveillance.

Beyond the clinic, plasmids play crucial roles in environmental adaptation. Research on the acidophile *Fervidacidithiobacillus caldus* identified over 30 plasmids and 50 defense systems, revealing an inverse correlation between plasmid diversity and host defense complexity (Pacheco-Acosta et al.). In cyanobacteria, novel replication proteins (Slr6031 and Slr6090) were discovered on large plasmids of *Synechocystis*, expanding the repertoire of Rep factors and opening avenues for genetic engineering (Ohdate et al.). These findings broaden our understanding of plasmid biology, showing that their roles extend far beyond resistance to shaping microbial ecology and providing tools for biotechnology.

From a technological perspective, new strategies for harnessing plasmids are also emerging. A systematic analysis of antimicrobial resistant isolates identified 22 conjugative plasmids with broad host potential, offering a toolbox for microbiome engineering and synthetic biology (Loyola Irizarry and Brito). In parallel, computational innovation is exemplified by plASgraph2, which employs graph neural networks to classify plasmid contigs from assembly graphs with high accuracy even on short sequences (Sielemann et al.). These contributions demonstrate how the integration of large-scale datasets with advanced algorithms can accelerate plasmid discovery and classification.

Collectively, the works in this Research Topic trace a trajectory: from urgent case studies of AMR pathogens, through eco-evolutionary perspectives, to computational and engineering frameworks. Each study illuminates a facet of plasmid diversity, while all converge on a central challenge—how to curate, annotate, and interlink plasmid data to build reliable, accessible, and comprehensive resources. Looking forward, the integration of expert curation, innovative machine learning, and experimental validation will be indispensable for transforming plasmid databases into true knowledge bases. Such efforts will not only strengthen public health surveillance and antimicrobial stewardship but will also open new opportunities in microbial ecology, biotechnology, and synthetic biology. Ultimately, advancing plasmid resources requires interdisciplinary collaboration across clinical microbiology, environmental microbiology, computational biology, and bioengineering, a collective endeavor to unlock the full potential of plasmids as both a global challenge and a global resource.
